# Monotonicity is a key feature of genotype-phenotype maps

**DOI:** 10.3389/fgene.2013.00216

**Published:** 2013-11-07

**Authors:** Arne B. Gjuvsland, Yunpeng Wang, Erik Plahte, Stig W. Omholt

**Affiliations:** ^1^Centre for Integrative Genetics (CIGENE), Department of Mathematical Sciences and Technology, Norwegian University of Life SciencesÅs, Norway; ^2^Centre for Integrative Genetics (CIGENE), Department of Animal and Aquacultural Sciences, Norwegian University of Life SciencesÅs, Norway; ^3^Department of Biology, Centre for Biodiversity Dynamics, NTNU Norwegian University of Science and TechnologyTrondheim, Norway

**Keywords:** genotype-phenotype map, gene regulatory networks, epistasis, variance component analysis, genetic modeling, systems genetics, genetic variance, monotonicity

## Abstract

It was recently shown that monotone gene action, i.e., order-preservation between allele content and corresponding genotypic values in the mapping from genotypes to phenotypes, is a prerequisite for achieving a predictable parent-offspring relationship across the whole allele frequency spectrum. Here we test the consequential prediction that the design principles underlying gene regulatory networks are likely to generate highly monotone genotype-phenotype maps. To this end we present two measures of the monotonicity of a genotype-phenotype map, one based on allele substitution effects, and the other based on isotonic regression. We apply these measures to genotype-phenotype maps emerging from simulations of 1881 different 3-gene regulatory networks. We confirm that in general, genotype-phenotype maps are indeed highly monotonic across network types. However, regulatory motifs involving incoherent feedforward or positive feedback, as well as pleiotropy in the mapping between genotypes and gene regulatory parameters, are clearly predisposed for generating non-monotonicity. We present analytical results confirming these deep connections between molecular regulatory architecture and monotonicity properties of the genotype-phenotype map. These connections seem to be beyond reach by the classical distinction between additive and non-additive gene action.

## Introduction

Quantitative genetics is the major theoretical foundation for genetic studies in production biology, evolutionary biology, and biomedicine. A core concept in quantitative genetics is the genotypic value, the mean observed phenotype for a given genotype. It constitutes the basis for the genotype-to-phenotype (GP) map concept. The shape of a given GP map is typically described by the classical gene action terms: additivity, dominance, and epistasis. Together with genotype frequencies in a given population, the GP map is the basis for decomposing observed phenotypic variance into environmental variance and genetic variance components including additive variance, dominance variance and epistatic variance. This provides the basis for a very successful theory when it comes to predicting selection response and breeding values (Falconer and Mackay, 1996; Lynch and Walsh, 1998) and more recent statistical genetics methods for mapping Quantitative Trait Loci (QTL) (Neale et al., 2008). Quantitative genetics thus provides a mature machinery for predicting the population level consequences of a given GP map, but in order to understand several generic genetic phenomena there is a stated need for new tools for disclosing how the shape of the GP map is determined by underlying biology (Jaeger et al., 2012; Moore, 2012; Gjuvsland et al., 2013).

One such phenomenon is the resemblance between parents and offspring. An explanation in quantitative genetic terms is that the additive variance (*V*_*A*_) makes up a substantial part of the phenotypic (*V*_*P*_) and genetic variance (*V*_*G*_). Hill et al. (2008) showed that in populations with extreme allele frequencies, high *V*_*A*_/*V*_*G*_ ratios will arise regardless of the shape of the GP map. However, for populations with intermediate allele frequencies a much wider range of *V*_*A*_/*V*_*G*_ ratios is observed (Wang et al., 2013). In such populations, high *V*_*A*_/*V*_*G*_ ratios cannot be fully accounted for without considering properties of the GP map. Gjuvsland et al. (2011) showed that a key feature of GP maps that give high ratios of additive to genotypic variance (*V*_*A*_/*V*_*G*_), is a monotone (or order-preserving) relation between gene content (the number of alleles of a given type) and phenotype. This led to the hypothesis that the regulatory circuitry of sexually reproducing organisms predominantly predisposes for highly monotone genotype-phenotype maps.

Here we address the above hypothesis by a two-step approach. First we provide methods and software tools for measuring monotonicity of generic GP maps (i.e., sets of genotypic values). Then we use these tools on the data generated by an extensive simulation study of a broad collection of gene regulatory network models. In these network models the steady state expression levels serve as phenotypes and genetic variation is introduced through parameters describing maximal production rates and the shape of the gene regulation function. Such *causally cohesive genotype-phenotype (cGP) models* [see Gjuvsland et al. (2013) and references therein] allow us to identify relationships between regulatory network architecture and properties of the resulting GP maps.

Our results confirm the prediction that the GP maps arising from a wide range of gene regulatory network motifs are in general highly monotone. In addition we show through numerical as well as mathematical analysis that regulatory motifs involving incoherent feed-forward or positive feedback stand out in their capacity to generate non-monotonicity. These relationships between molecular regulatory architecture and properties of the genotype-phenotype map—of substantial relevance to functional genomics in general—are beyond reach by the standard distinction between additive and non-additive gene action.

Our approach can be applied to cGP models of a wide range of biological systems at any level of model complexity. It opens for a systematic study of the monotonicity properties of molecular regulatory structures underlying the whole spectrum of physiological regulation. This suggests that the concept of monotonicity of GP maps can be used to build theory about heredity phrased in terms of molecular mechanism, something which standard genetic concepts and approaches appear to be incapable of.

## Models and methods

### Background on monotonicity of GP maps

To ease understanding we provide a brief recapitulation of the concept of monotonicity (or order-preservation) in GP maps introduced in (Gjuvsland et al., 2011). We consider a diploid genetic model with *N* biallelic loci (alleles indexed 1 and 2) underlying a quantitative phenotype. A genotype at a single locus *k* is denoted by *g*_*k*_ ∈ {11, 12, 22}. In the case of two loci *k* and *l* there are 9 possible genotypes *g*_*kl*_ = *g*_*k*_*g*_*l*_ ∈ {1111, 1112, 1122, 1211, …, 2212, 2222}. The general *N* loci genotype space Γ contains 3^*N*^ genotypes *g*_1_*g*_2_ … *g*_*N*_ (in condensed notation *g*_1:*N*_) constructed by concatenating single locus genotypes, Γ = {*g*_1_*g*_2_ … *g*_*N*_ | *g*_*k*_ ∈ {11, 12, 22}, *k* = 1, 2, …, *N*}. For any locus *k, the genotypic background*, i.e., the allele composition of all loci *except k*, is *g*^(*k*)^ = *g*_1_*g*_2_ … *g*_*k* − 1_ *g*_*k* + 1_ … *g*_*N*_ = *g*_1: *k* − 1_ *g*_*k* + 1: *N*_. For example, if *N* = 4 then *g*^(2)^ = 112212 means that the genotypes of locus 1, 3, and 4 are 11, 22, and 12, respectively. We use the straightforward notation *g*_1_ *g*_2_ … *g*_*k* − 1_ 11*g*_*k* + 1_ … *g*_*N*_ = *g*_1:*k* − 1_ 11*g*_*k* + 1: *N*_ to indicate a genotype where *g*_*k*_ = 11 while the background genotype is arbitrary. We will also use the compressed notation 11*g*^(*k*)^ (or generally *g*_*k*_*g*^(*k*)^).

**Figure 1:**
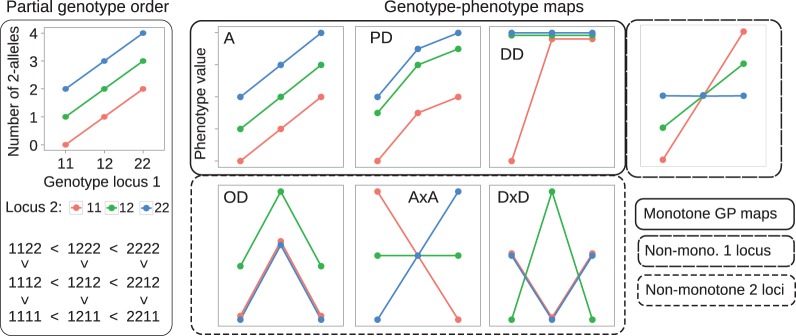
**Examples of partial genotype order and genotype-phenotype maps**. **Left panel**: The allele content defines a partial order on genotype space. A two-locus example is shown. The plot at the top displays the genotype at locus 1 (x-axis) and locus 2 (color) vs. the total number of 2-alleles (y-axis) in the two-locus genotype. The resulting partial ordering of genotypes is shown below. **Right panel:** Each lineplot shows the 9 genotypic values (y-axis) for a single GP map, coding of genotype are the same as in the left panel. GP maps that preserve the partial order of genotypes are called monotone. Examples shown are an intra- and interlocus additive map (A), a map showing partial dominance at both loci (PD), and duplicate dominant (DD) epistasis (see Table 1 in Phillips, 1998). GP maps that break the partial order of genotypes are called non-monotone, examples shown are pure overdominance at both loci (OD), additive-by-additive epistasis (A × A) and dominance-by-dominance epistasis (D × D). The rightmost plot shows a GP map that is monotone w.r.t. locus 1, but non-monotone w.r.t. locus 2.

We use the 2-allele content (i.e., the number of 2-alleles) of genotypes to define a partial order on the genotype space Γ (see Figure 1, left panel for an illustration). For a particular locus *k* we order the three genotypes sharing the same background genotype *g*_1: *k* − 1_ *g*_*k* + 1: *N*_ as follows,

(1) $$g_{1:k-1}11g_{k+1:N}<g_{1:k-1}12g_{k+1:N}<g_{1:k-1}22g_{k+1:N}$$

We call this the *partial genotype order relative to locus k*, and it defines a strict partial order on Γ.

A genotype-phenotype map is a mapping *G* that assigns to each genotype *g* ∈ Γ a real-valued genotypic value *G*(*g*) (the mean trait value for a given genotype). We define monotonicity of *G* in terms of how it transforms the partial genotype order to the algebraic order of the genotypic values *G*(*g*). Without loss of generality we assume that the allele indexes at each locus have been chosen such that *G*(1111 … 11) is the smallest of all homozygote genotypic values. We call a genotype-phenotype map *G monotone or order-preserving with respect to locus k* if it preserves the partial genotype order relative to locus *k*, i.e., if,

(2) $$\begin{array}{l} G(g_{1:k-1}11g_{k+1:N})\leq G(g_{1:k-1}12g_{k+1:N})\\ \;\leq G(g_{1:k-1}22g_{k+1:N}) \end{array}$$

for all genetic backgrounds of locus *k*. By allowing non-strict inequalities we include GP maps showing complete dominance and complete magnitude epistasis (Weinreich et al., 2005) in the class of order-preserving GP maps. Conversely we call a GP map *non-monotone* or *order-breaking with respect to locus k* if it does not preserve the partial genotype order relative to locus *k* for all backgrounds. Figure 1 (right panel) shows classical dominance and epistasis patterns, categorized into monotone and non-monotone GP maps.

### Statistical decomposition of genotype-phenotype maps

Given a genotype-phenotype map *G* as described above and a corresponding vector of genotype frequencies *f* in a population, quantitative genetic provides methods for orthogonal decomposition of genotypic values and resulting genetic variance in the population into additive and non-additive (dominance and epistasis) components (Lynch and Walsh, 1998). We performed such statistical decomposition with the function linearGPmapanalysis in the R package noia (http://cran.r-project.org/package=noia; Le Rouzic and Alvarez-Castro, 2008) version 0.94.1. We assumed an idealized population where all genotype frequencies are equal (1/3^*N*^). In such a hypothetical population the NOIA (Alvarez-Castro and Carlborg, 2007) statistical and functional formulations and the unweighted regression model proposed by Cheverud and Routman (1995) are equivalent. Furthermore, the decomposition of genotypic values is equivalent to decomposing *G* into a sum of additive and non-additive GP maps, and the genetic variance in this case is simply the variance of the 3^*N*^ genotypic values in *G*. We used the NOIA statistical formulation to decompose a GP map *G* into its additive and non-additive components, and computed the ratio of additive to total genetic variance *V*_*A*_/*V*_*G*_ as a measure of how well the additive component describes the original GP map. In case of the illustrative GP maps depicted in Figure 1, this gives *V*_*A*_/*V*_*G*_ = 1 for the fully additive GP map A, and *V*_*A*_/*V*_*G*_ = 0 for the pure overdominance (OD) and the pure epistasis (Cheverud and Routman, 1996) maps A × A and D × D.

### Gene regulatory network models

Gene expression in eukaryotes is controlled through gene regulatory networks involving numerous regulatory mechanisms [see e.g., Latchman (2005), for details]. Modeling of such gene regulatory networks is well-established, and available modeling frameworks range from coarse-grained descriptions of the topology of genome-wide networks to very detailed mechanistic models describing the dynamics of small networks (De Jong, 2002; Schlitt and Brazma, 2007; Karlebach and Shamir, 2008). In line with a large number of authors we used ordinary differential equations (ODEs) to study a family of generic gene regulatory network models containing three diploid genes *X*_1_, *X*_2_, and *X*_3_, organized as a regulatory system where the rate of expression of each gene can be regulated by the expression level of one or both of the other genes. The wiring of the system is described by a 3 × 3 connectivity matrix *A* with elements *A*_*kl*_ ∈ {−1, 0, 1}. The signs of the elements of *A* describe the mode of regulation, *A*_*kl*_ = 0 indicates that *X*_*l*_ is not a regulator of *X*_*k*_, if *A*_*kl*_ = 1 then *X*_*l*_ is an activator of *X*_*k*_, and if *A*_*kl*_ = −1 then *X*_*l*_ is a repressor of *X*_*k*_. Gene regulatory systems are often laid out visually as signed directed graphs. There is a one-to-one correspondence between a connectivity matrix and a signed directed graph, two examples are illustrated in Figure 4. We used the sigmoid formalism (Mestl et al., 1995; Plahte et al., 1998) in the diploid form (Omholt et al., 2000) where the expression the two alleles of gene *k* is described by the following ODEs,

(3) $$\begin{array}{l} {\dot{x}}_{k1}=\alpha_{k1}R_{k1}(y_{1},y_{2},y_{3})-\gamma_{k1}x_{k1},\\ {\dot{x}}_{k2}=\alpha_{k2}R_{k2}(y_{1},y_{2},y_{3})-\gamma_{k2}x_{k2},\\ \;y_{k}=x_{k1}+x_{k2},\;k=1,2,3. \end{array}$$

Here α_*ki*_ is the maximal production rate for allele *i* of gene *X*_*k*_, γ_*ki*_ is the decay rate, while *R*_*ki*_ is the gene regulation function (dose-response function). If *X*_*k*_ has no regulators, we assume production is always switched on i.e., *R*_*ki*_ = 1. If *X*_*k*_ has a single regulator *X*_*l*_, the gene regulation function is given as *R*_*ki*_(*y*_*l*_) = *S*(*y*_*l*_, θ_*lki*_, *p*_*lki*_), where *S*(*y*, θ, *p*) = *y*^*p*^/(*y*^*p*^ + θ^*p*^) if *X*_*l*_ is an activator and *S*(*y*, θ, *p*) = 1 − *y*^*p*^/(*y*^*p*^ + θ^*p*^) if it is a repressor. In both cases the parameter θ_*lki*_ gives the amount of regulator needed to get 50% of maximal production rate, and *p*_*lki*_ determines the steepness of the response. In the case of two regulators *X*_*l*_ and *X*_*j*_ we set *R*_*ki*_(*y*_*l*_, *y*_*j*_) = *S*(*y*_*l*_, θ_*lki*_, *p*_*lki*_)*S*(*y*_*j*_, θ_*jki*_, *p*_*jki*_), corresponding to the Boolean AND function. Modeling transcription regulation by means of Hill functions and Boolean composition has a long tradition in modeling of gene regulation and is widely used.

With three genes and up to two regulators per gene the number of possible connectivity matrices is 6859. We further required that the system is connected, and that *X*_3_ is downstream to both *X*_1_ and *X*_2_ so either *X*_1_ and *X*_2_ both regulate *X*_3_ directly (*A*_31_*A*_32_ ≠ 0), or one of them regulates *X*_3_ directly and the other one indirectly (*A*_31_*A*_12_ ≠ 0 or *A*_32_*A*_21_ ≠ 0). This reduces the number of distinct connectivity matrices to 3724. Finally, we identified pairs of matrices that are symmetric with respect to interchanging *X*_1_ and *X*_2_ and picked just one matrix from each pair. The resulting 1881 connectivity matrices were used for our gene regulatory simulations.

### Identifying feedback loops and feedforward motifs

Feedback and feedforward motifs appear recurrently as regulatory building blocks in transcription networks across all living organisms. These network motifs have several characteristic features (Alon, 2007), negative feedback can for example accommodate fast transcriptional responses and homeostasis, while positive feedbacks are utilized as biological switches. We went through all 1881 gene regulatory models and extracted information about their feedback and feedforward loop characteristics from their connectivity matrices. For each system we computed three autoregulatory feedback loop products *FL*_1_ = *A*_11_, *FL*_2_ = *A*_22_, *FL*_3_ = *A*_33_, three two-gene feedback loop products: *FL*_12_ = *A*_21_*A*_12_, *FL*_13_ = *A*_31_ *A*_13_, *FL*_23_ = *A*_23_ *A*_32_ and two three-gene feedback loop products: *FL*_123_ = *A*_32_ *A*_21_ *A*_13_, *FL*_213_ = *A*_31_ *A*_12_*A*_23_. Non-zero loop products indicate that the system contains the corresponding feedback loop, and the sign of the loop product gives the sign of the feedback loop. We also computed the products for two feedforward motifs: *FFL*_32_ = *A*_32_(*A*_31_ *A*_12_), *FFL*_31_ = *A*_31_ (*A*_32_*A*_21_). Again non-zero products indicate that the system contains the corresponding feedforward motif, a positive value corresponds to a coherent feedforward while a negative value indicates incoherent feedforward. Figure 4 depicts the connectivity matrix and the signed digraphs of a system with a positive feedback loop as well as a system with incoherent feedforward. Spreadsheet S1 contains adjacency matrices and loop products for all 1881 motifs.

### Gene regulatory network simulations

The simulation were performed with the Python package cgptoolbox (http://github.com/jonovik/cgptoolbox), using the sigmoidmodel submodule, which contains an implementation of the gene regulatory network model (Equation 3) and the connectivity matrix *A*. A similar simulation setup is found in Gjuvsland et al. (2011) together with a discussion of gene regulation functions and the genotype-parameter map in molecular terms. We compared two different types of genotype-to-parameter maps:

- *Genotype to parameter map without pleiotropy:* biallelic genotypic variation for all three loci was introduced through the maximal production rates α_*ki*_. For each Monte Carlo simulation the allelic parameter values were sampled from *U*(100, 200).
- *Genotype to parameter map with pleiotropy:* allelic parameter values were sampled for maximal production rates α_*ki*_ (sampled from *U*(100, 200)), regulation thresholds θ_*lki*_ (sampled from *U*(20, 40)), and regulation steepnesses *p*_*lki*_ (sampled from *U*(1, 10)).

All decay rates γ_*ki*_ were set equal to 10. We assembled parameter sets for all 27 diploid genotypes, and for each genotypic parameter set the system of Equation 3 was integrated numerically until convergence to a stable state. The equilibrium value of *y*_3_ was recorded as phenotype. Datasets where the system failed to converge for one or more genotypes were discarded. For each of the 1881 motifs we performed 1000 Monte Carlo simulations.

Some Monte Carlo simulations lead to very little phenotypic variation, in the sense that the span between the largest and smallest of the 27 genotypic values was small. In order to avoid artifacts arising from the numeric ODE solver tolerance, these essentially flat GP maps were discarded. Further analysis of monotonicity and variance components were only performed on GP maps where the absolute range (maximum genotypic value – minimum genotypic value) and relative range (absolute range/mean genotypic value) were both > 0.01.

## Results

### Measuring monotonicity of GP maps

In the following we present two numerical measures for quantifying monotonicity in a GP map *G* with *N* biallelic loci. The first quantifies the monotonicity for individual loci by comparing negative and positive allele substitution effects before weighting the individual loci into an overall measure. The second utilizes isotonic regression to quantify the distance between *G* and the closest fully monotone GP map.

#### Measure 1: quantifying non-monotonicity by substitution effects

We first develop a measure of monotonicity based on the effects of substituting a single allele at locus *k*,

(4) $$\begin{array}{l} s^{1}(g^{\left(k\right)})=G(g_{1:k-1}22g_{k+1:N})-G(g_{1:k-1}12g_{k+1:N}),\\ s^{2}(g^{\left(k\right)})=G(g_{1:k-1}12g_{k+1:N})-G(g_{1:k-1}11g_{k+1:N}), \end{array}$$

while keeping the background genotype *g*^(*k*)^ = *g*_1: *k* + 1_*g*_*k* + 1: *N*_ fixed. Monotonicity as defined by Equation 2 is equivalent to *s*^*i*^ (*g*^(*k*)^) ≥ 0 for *i* = 1, 2 across all genetic backgrounds of locus *k*. By taking into account also the magnitude of the substitution effects we can quantify the deviation from strict monotonicity. We start with the set *S*^*k*^ = {*s*^*i*^(*g*^(*k*)^)} of single allele substitution effects for locus *k* for *i* = 1, 2 and across all genotypic backgrounds *g*^(*k*)^. The total number of elements in *S*^*k*^ thus becomes 2· 3^*N* − 1^, and we split the set into two disjoint subsets reflecting their sign; *S*^*k*^_+_ = {*s*^*i*^(*g*^(*k*)^) ∈ *S*^*k*^ | *s*^*i*^ (*g*^(*k*)^) > 0} and *S*^*k*^_−_ = {*s*^*i*^ (*g*^(*k*)^) ∈ *S*^*k*^ | *s*^*i*^ (*g*^(*k*)^) < 0}. We compute the sum of positive substitution effects and the sum of absolute values of negative substitution effects,

(5) $$P_{k}=\sum_{S_{+}^{k}}s^{i}(g^{\left(k\right)}),$$

$$N_{k}=\sum_{S_{-}^{k}}\left|s^{i}(g^{\left(k\right)})\right|,$$

and let *T*_*k*_ = *P*_*k*_ + *N*_*k*_ denote the overall sum of absolute substitution effects. We then define the degree to which the GP map *G* is monotone with respect to locus *k* by,

(6) $$m_{k}=\frac{\left|P_{k}-N_{k}\right|}{T_{k}}=\frac{\left|\sum_{g^{\left(k\right)}}\left(s^{1}(g^{\left(k\right)})+s^{2}(g^{\left(k\right)})\right)\right|}{\sum_{g^{\left(k\right)}}\left(\left|s^{1}(g^{\left(k\right)})|+|s^{2}(g^{\left(k\right)})\right|\right)}.$$

The absolute value in the numerator ensures that the measure *m*_*k*_ is invariant with respect to the choice of indexes for the two alleles of locus *k*. Interchanging the numbering of the alleles leads to the mappings *s*^1^ (*g*^(*k*)^) ↦ −*s*^2^(*g*^(*k*)^), *s*^2^(*g*^(*k*)^) ↦ −*s*^1^ (*g*^(*k*)^), which leaves the value of *m*_*k*_ unchanged. By the triangle inequality *m*_*k*_ ≤ 1. If *m*_*k*_ = 1, then *G* is monotonic with respect to locus *k*, whereas *m*_*k*_ < 1 implies that *G* is order-breaking w.r.t. locus *k*. If *m*_*k*_ = 0, then the positive substitution effects equal the negative substitution effects in magnitude and we say that *G* is completely order-breaking w.r.t. locus *k*. This measure distinguishes well between the monotone and non-monotone maps in Figure 1. Clearly *m*_1_ = *m*_2_ = 1 for the additive map (A) and GP maps showing partial dominance and duplicate dominance epistasis. In contrast, *m*_1_ = *m*_2_ = 0 for the maps showing pure OD and pure epistasis (A × A and D × D).

In order to quantify the overall monotonicity of the GP map *G* we introduce the *degree of monotonicity (m)* which is a weighted mean of all *m*_*k*_, where the weights reflect the relative effect size of the loci in terms of *T*_*k*_,

(7) $$m=\frac{\sum_{k=1}^{N}m_{k}T_{k}}{\sum_{k=1}^{N}T_{k}}.$$

As shown in Figure 3A, the *degree of monotonicity* is accordingly 1 for the monotone maps in Figure 1 while it is 0 for the pure OD and pure epistasis maps. This definition of *degree of monotonicity* allows us to establish a vocabulary that is analogous to the classification of single locus dominance; i.e., a GP map is called *monotone* if *m* = 1, (*partially) non-monotone* if *m* < 1 and *purely non-monotone* if *m* = 0.

**Figure 2:**
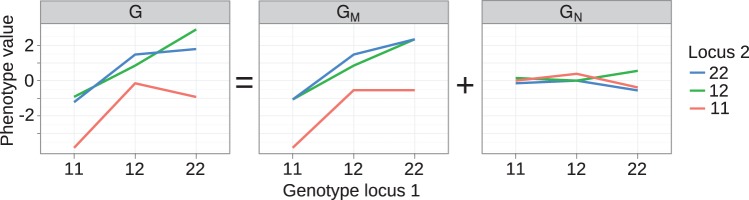
**Decomposition of genotype-phenotye map into monotone and non-monotone components**. **Left panel:** Genotype-phenotype map *G* for two loci underlying 10-week body-weight at 10 weeks in a mouse *F*_2_ cross. The GP map shown here is equivalent to the one in the original publication [see Figure 3A in Cheverud and Routman (1995)], but we have changed indexing of loci and alleles for consistency with the notation used here. The GP map *G* is decomposed with isotonic regression into a **(middle panel)** monotone component *G*_*M*_ and a (**right panel**) non-monotone component *G*_*N*_.

For example, the degree of monotonicity of the GP map published by Cheverud and Routman (1995), with two loci underlying 10-week body-weight (in grams) at 10 weeks in a mouse *F*_2_ cross, may be computed as follows. After renaming the two loci (B → 1, A → 2) and indexing alleles to conform to our notation, the nine genotypic values (Table 1 in (Cheverud and Routman, 1995)) are *G*(1111) = 31.23, *G*(1112) = 34.13, *G*(1122) = 33.82, *G*(1211) = 34.89, *G*(1212) = 35.90, *G*(1222) = 36.53, *G*(2211) = 34.12, *G*(2212) = 37.95, and *G*(2222) = 36.84. From the line plot of this GP map (Figure 2, left panel) we find that the GP map is non-monotone with respect to both loci. Locus 1 shows marginal OD for the 11 genotype of locus 2 and locus 2 shows marginal OD for the 11 and 22 genotypes of locus 1. To compute the degree of monotonicity, we start with the set of single allele substitution effects for locus 1, *S*^1^ = {3.66, −0.77, 1.77, 2.05, 2.71, 0.31}, and divide this into sets of negative *S*^1^_−_ = {−0.77} and positive effects *S*^1^_+_ = {3.66, 1.77, 2.05, 2.71, 0.31}. The sum *N*_1_ of elements in *S*^1^_+_ is 10.50 and *P*_1_ the sum of absolute values of elements in *S*^1^_−_ is 0.77, which gives *T*_1_ = *P*_1_ + *N*_1_ = 11.27. From Equation 6 it follows that *m*_1_ = 0.86. Similarly, the sets of substitution effects for locus 2 are *S*^2^_−_ = {−1.11, −0.31} and *S*^2^_+_ = {3.83, 0.63, 1.01, 2.90}. This gives, *N*_2_ = 1.42, *P*_2_ = 8.37, *T*_2_ = 9.79, and *m*_2_ = 0.71. Inserting values for both loci into Equation 7, the degree of monotonicity (*m*) of this GP map is calculated to be 0.79. This value concords well with the visual observation (Figure 2, left panel) that it does not deviate substantially from a purely monotone map.

**Figure 3:**
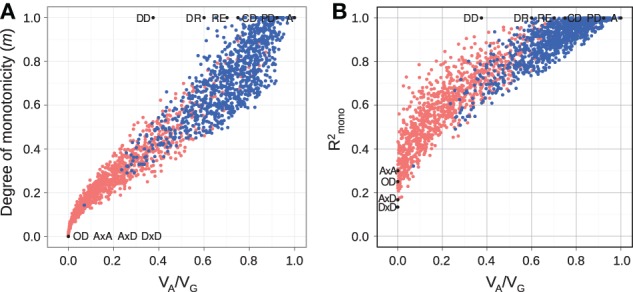
**Measures of monotonicity vs. additivity of GP maps**. Scatterplots showing *V*_*A*_/*V*_*G*_ from unweighted regression vs. **(A)** degree of monotonicity (*m*) and **(B)** *R*^2^_mono_ from isotonic regression. Black dots correspond to the maps shown in Figure 1 together with additive-by-dominance epistasis (A × D), a map with two loci showing complete dominance (CD) and two classical epistasis types from Table 1 in Phillips (1998); duplicate recessive genes (DR) and recessive epistasis (RE). Red dots show 1000 random two-locus GP maps, while blue dots show the same 1000 GP maps after rearranging genotypic values to introduce order-preservation for 1 locus [see Model and Methods in Gjuvsland et al. (2011)].

**Figure A1:**
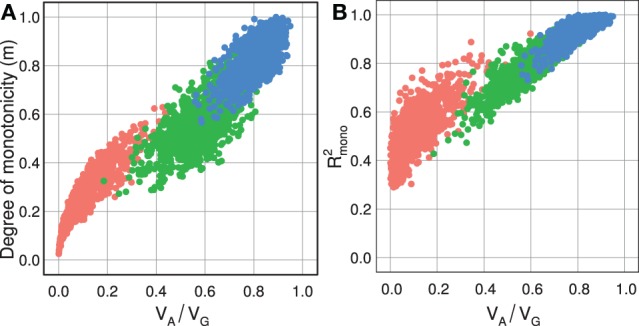
**Measures of monotonicity vs. additivity of GP maps with three loci**. Scatterplots showing *V*_*A*_/*V*_*G*_ from unweighted regression vs. **(A)** degree of monotonicity (*m*) and **(B)** *R*^2^_mono_. Red dots show 1000 random three-locus GP maps, blue dots show the same 1000 GP maps after sorting to introduce order-preservation for 1 locus while green dots show the same 1000 GP maps after sorting to introduce order-preservation for 2 loci [see Model and Methods in Gjuvsland et al. (2011)].

For random GP maps (randomly sampled genotypic values as in (Gjuvsland et al., 2011)) there is a strong positive correlation between the degree of monotonicity and the size of the additive component (*V*_*A*_/*V*_*G*_) (Figure 3A). A similar relationship was observed for three-locus random GP maps (Figure A1A). All GP maps in Figure 3A with *m* < 0.1 have *V*_*A*_/*V*_*G*_ < 0.1. At the other end of the spectrum there is much more variation, for instance the most extreme completely monotone map (the duplicate dominant factors DD) has *V*_*A*_/*V*_*G*_ as low as 0.375.

#### Measure 2: quantifying monotonicity by isotonic regression

This measure quantifies the monotonicity of a particular GP map *G* in terms of the least-squares distance to the closest monotone map. We build on the mathematical notation introduced in section “Background on monotonicity of GP maps” where Γ is the genotype space for *N* biallelic loci and a GP map is a function that assigns a real-valued genotypic value *G*(*g*) to each genotype *g* in Γ. For any particular GP map *G*, we identify the *monotone component* of *G* as the map *G*_*M*_ which minimizes the residual variance var(*G* − *G*_*M*_), i.e., *G*_*M*_ is the monotone GP map which is closest to *G* in the least-squares sense. For a given *G* the monotone component *G*_*M*_ is unique (Barlow and Brunk, 1972) and can be computed numerically by isotonic regression (Leeuw et al., 2009) of *G* subject to the partial ordering of genotypes defined in Equation 1. Furthermore, the residual *G*_*N*_ is orthogonal to *G*_*M*_ in the sense that ∑_*g* ∈ Γ_ *G*_*M*_ (*g*)*G*_*N*_ (*g*) = 0. This allows the orthogonal decomposition,

(8) $$G=G_{M}+G_{N},$$

of a genotype-phenotype map into a *monotone component G*_*M*_ and a *non-monotone component G*_*N*_ such that var(*G*) = var(*G*_*M*_) + var(*G*_*N*_). The orthogonality property allows us to measure monotonicity of *G* in terms of the coefficient of determination *R*^2^_mono_ of the isotonic regression given by the ratio *R*^2^_mono_ = var(*G*_*M*_)/var(*G*). In the case that *G* itself is monotone for all loci we have *R*^2^_mono_ = 1, while order-breaking for one or more loci will result in *R*^2^_mono_ < 1.

The isotonic regression approach can be illustrated in a straightforward way on the two-locus GP map provided by Cheverud and Routman (1995) (see text above and left panel of Figure 2). The partial ordering of genotypes defined by Equation 1 is illustrated in Figure 1 (left panel). By isotone regression (Leeuw et al., 2009) on this partial genotype ordering, the original GP map is decomposed into a monotone and a non-monotone component (Figure 2, middle and right panels), and the coefficient of determination (*R*^2^_mono_) is 0.97.

**Figure A2:**
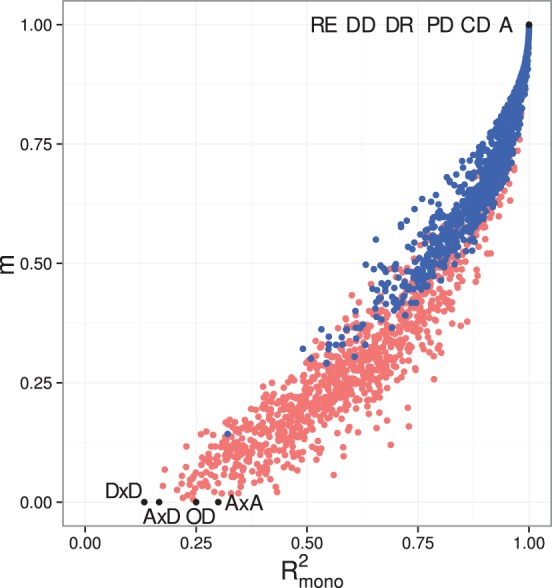
**Comparing measures of monotonicity GP maps**. Scatterplots showing degree of monotonicity (*m*) vs. *R*^2^_mono_. Black dots correspond to the maps shown in Figure 1. Red dots show 1000 random two-locus GP maps, while blue dots show the same 1000 GP maps after sorting to introduce order-preservation for 1 locus [see Model and Methods in Gjuvsland et al. (2011)].

Our simulation results for random GP maps show that *R*^2^_mono_ is positively correlated to the size of the additive component (Figure 3B for two-locus GPs maps and Figure A1B for three-locus GP maps) and that for a given *V*_*A*_/*V*_*G*_ the lower bound for *R*^2^_mono_ is close to a straight line from (0, 0.2) to (1, 1). However, due to the search for the closest monotone GP map, *R*^2^_mono_ will not become zero even for purely overdominant or purely epistatic maps. As shown in Figure A2, the two monotonicity measures are highly correlated.

#### An R package for studying monotonicity in GP maps

We developed an R package gpmap for studying functional properties of GP maps. The package takes GP maps in the form of vectors of genotypic values as input, and provides functions for (i) determining whether the map is order-breaking or order-preserving w.r.t. any given locus, (ii) the degree of monotonicity *m*, (iii) *R*^2^_mono_ using isotonic regression from the isotone package (Leeuw et al., 2009), and (iv) plots of the original and decomposed GP maps. Code example 1 (Box 1) below illustrates the usage and functionality of the gpmap package. The package is available from CRAN http://cran.r-project.org/package=gpmap under GPLv3.

Box 1 **Code example 1.**

Code example for quantifying and visualizing monotonicity for the two-locus GP map published in [14] using the R package gpmap.


> library(gpmap) #load package
> data(GPmaps) #load dataset
> gp <- mouseweight #GP map from reference
  [14]
>
> ## Tabulate genotypic values
> cbind(gp$genotype,gp$values)
>
> ## Plot the GP map
> plot(gp)
>
> ## Compute degree of monotonicity
> gp <- degree_of_monotonicity(gp)
> gp$degree.monotonicity.locus
> print(gp)
>
> ## Quantify monotonicity by isotonic
  regression
> gp <- decompose_monotone(gp)
> print(gp)
>
> ## Plot decomposed GP map
> plot(gp,decomposed=TRUE)

### Monotonicity in GP maps arising from gene regulatory networks

**Figure 4:**
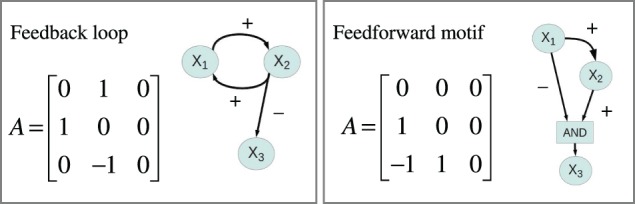
**Connectivity matrices and signed directed graphs**. Connectivity matrix *A* and the corresponding signed directed graph for two of the 1881 systems in the simulation study. The **left panel** depicts the connectivity matrix and the signed digraph of a system with a positive feedback loop between *X*_1_ and *X*_2_ while the **right panel** shows a system with incoherent feedforward from *X*_1_ to *X*_3_.

To search for generic relationships between monotonicity and regulatory network structure, we used the above measures of monotonicity to characterize GP maps emerging from the gene regulatory network models (see Models and Methods). Based on earlier results (Gjuvsland et al., 2007, 2011; Wang et al., 2013) we hypothesized that incoherent feed forward (Figure 4, right panel) or positive feedback (Figure 4, left panel) would be necessary in order to obtain highly order-breaking GP maps, and we characterized all 1881 networks in terms of these two properties. Table 1 shows the number of motifs falling into the resulting four categories. We summarized the number of Monte Carlo simulations where all genotypic parameter sets gave convergence to a stable steady state, and where the resulting GP maps were not essentially flat (see Models and Methods for details). Motifs with less than 100 usable GP maps were discarded from further analysis. For the genotype-to-parameter maps without pleiotropy (in the sense that genetic variation at one locus influences only a single parameter, see Model and Methods) 868 motifs were discarded, while for the genotype-to-parameter map with pleiotropy (genetic variation at one locus influences three parameters) 791 motifs were discarded. All (but one) discarded motifs contained at least one positive feedback loop (Table 1). A plausible explanation for this is that many motifs with positive feedback loops have a stable steady state at, or very close to 0 for one or more state variables regardless of parameter values, and this leads to essentially flat GP maps.

**Table 1:** **Frequencies (proportion of row total in parenthesis) of incoherent feedforward and positive feedback loops in subsets of the 1881 studied motifs**.

**Dataset**	**Number of motifs**	**Motifs containing**			
		**Incoh. feedforward**		**No incoh. feedforward**	
		**Positive feedback**	**No positive feedback**	**Positive feedback**	**No positive feedback**
All motifs	1881	287 (0.153)	48 (0.026)	1294 (0.688)	252 (0.134)
**GENOTYPE-TO-PARAMETER MAP WITHOUT PLEIOTROPY**					
Discarded motifs	868	152 (0.175)	0	715 (0.824)	1 (0.001)
Analyzed motifs	1013	135 (0.133)	48 (0.047)	579 (0.571)	251 (0.248)
**GENOTYPE-TO-PARAMETER MAP WITH PLEIOTROPY**					
Discarded motifs	791	124 (0.157)	0	667 (0.84)	0
Analyzed motifs	1090	163 (0.149)	48 (0.044)	627 (0.575)	252 (0.231)

**Figure 5:**
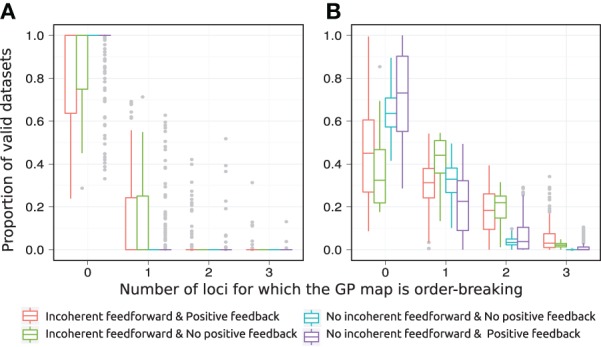
**Order-breaking in motifs containing a single feedforward loop**. Summary of order-breaking for all motifs for which at least 100 (out of 1000) Monte Carlo simulations lead to GP maps with non-negligible variation (see Models and Methods section “Gene regulatory network simulations,” for detailed criteria). Results are shown for 1013 motifs with a genotype-to-parameter map without pleiotropy **(A)** and 1090 motifs with a genotype-to-parameter map with pleiotropy **(B)**. Colors indicate classes of motifs based on the presence/absence of incoherent feedforward and positive feedback loops, see Table 1 for the number of motifs in each class. A single boxplot summarizes, for all motifs in the given class, the proportion of the GP maps (y-axis) that are order-breaking with respect to a given number of loci (x-axis). For example, consider the red box at *x* = 0 in panel **(A)**. This boxplot contains results for motifs with both incoherent feedforward and positive feedback and from Table 1 we find that the red boxplot summarizes results for 135 motifs. From the y-axis we find that at least half (box median at *y* = 1) of these 135 motifs result in only monotone GP maps, while for the most extreme (end of whisker) of the 135 motifs only 25% of the GP maps are monotone. Similarly, the cyan box is compressed into a line at *x* = 0, *y* = 1 indicating that all 251 motifs that lack both incoherent feedforward and positive feedback result in only monotone GP maps.

The introduction of pleiotropy in the genotype to parameter map has a marked effect on the monotonicity characteristics of the associated GP map (Figure 5). When genetic variation at a locus *X*_*i*_ affects only its maximal production rate the GP maps come out as highly monotone (Figure 5A), with a large majority being fully monotone or order-breaking for just a single locus. When genetic variation at locus *X*_*i*_ affects the threshold and steepness of the dose-response curve in addition to the maximal production rate (pleiotropy in the genotype-to-parameter map), the majority of GP maps still show order-breaking either for no loci or just one locus (Figure 5B). But a considerable number of GP maps are in this case order-breaking for two or three loci. Furthermore, by dividing the motifs into the four groups given in Table 1 it is evident that the regulatory anatomy of a network determines its predisposition for non-monotonicity in its associated GP map. Presence of incoherent feedforward or positive feedback loops appears to be prerequisites for the majority of the observed non-monotonic GP maps.

The class of motifs lacking both incoherent feedforward and positive feedback contains very few order-breaking GP maps, and with no pleiotropy in the genotype-to-parameter map we observe only fully order-preserving GP maps for this class (cyan in Figure 5A). In the Appendix we generalize this to an arbitrary number of nodes and formally prove that without pleiotropy in the genotype-to-parameter map, the presence of incoherent feedforward or positive feedback is indeed a necessary condition for non-monotone GP maps to arise from networks with monotone gene regulation functions.

**Figure 6:**
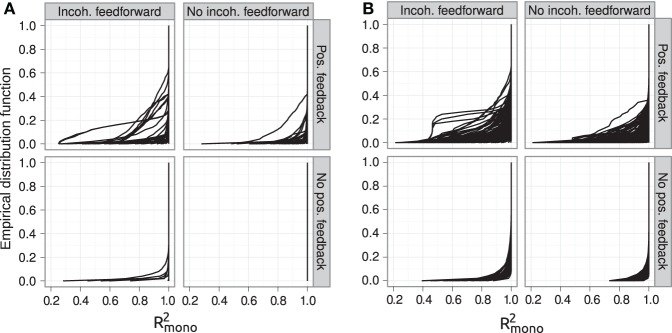
**Empirical distribution functions for *R*^2^_mono_**. Summary of *R*^2^_mono_ values from isotone regression for all motifs for which at least 100 (out of 1000) Monte Carlo simulations lead to GP maps with non-negligible phenotypic variation (see Models and Methods section “Gene regulatory network simulations,” for detailed criteria). Results are shown for 1013 motifs with a genotype-to-parameter map without pleiotropy **(A)** and 1090 motifs with a genotype-to-parameter map with pleiotropy **(B)**. Each panel is divided into 4 subplots containing classes of motifs based on the presence/absence of incoherent feedforward and positive feedback loops, see Table 1 for the number of motifs in each class. Each curve shows, for a single motif, the empirical distribution function value (y-axis) of *R*^2^_mono_ for all GP maps (x-axis).

**Figure A3:**
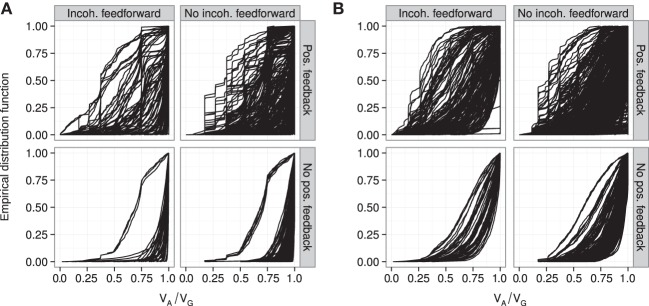
**Empirical distribution functions for additivity of GP maps**. Summary of *V*_*A*_/*V*_*G*_ from unweighted regression for all motifs for which at least 100 (out of 1000) Monte Carlo simulations lead to GP maps with non-negligible phenotypic variation (see Models and Methods section “Gene regulatory network simulations,” for detailed criteria). Results are shown for 1013 motifs with a genotype-to-parameter map without pleiotropy **(A)** and1090 motifs with a genotype-to-parameter map with pleiotropy **(B)**. Each panel is divided into 4 subplots containing classes of motifs based on the presence/absence of incoherent feedforward and positive feedback loops, see Table 1 for the number of motifs in each class. Each curve shows, for a single motif, the empirical distribution function value (y-axis) of *V*_*A*_/*V*_*G*_ from unweighted regression for all GP maps (x-axis).

The introduction of pleiotropy in the genotype-to-parameter map increases the frequency of order-breaking GP maps substantially (Figure 5B). Motifs lacking both incoherent feedforward and positive feedback may in this case lead to GP maps that are order-breaking for one or two loci, but never for all three loci. Using isotonic regression to quantify the overall monotonicity of the GP maps reinforces the finding that incoherent feedforward and positive feedback predispose for non-monotonicity (Figure 6). Figure 6 also shows that for all classes of motifs the majority of GP maps are fully monotone, while the most non-monotone GP maps (lowest *R*^2^_mono_ values) are observed for motifs with positive feedback. The differences between classes of motifs are also evident when inspecting the additivity of GP maps (Figure A3), but since monotone GP maps can still be non-additive, the patterns are much more blurred than for monotonicity.

## Discussion

Fisher's (1918) regression on gene content and the concepts derived from this, such as additive effects and dominance deviation, provide the theoretical basis for most of quantitative genetics (Falconer and Mackay, 1996; Lynch and Walsh, 1998). By regressing on gene content, including the extensions by Cockerham (1954), the genotype-phenotype map is decomposed into additive, dominant, and epistatic components. The use of gene content or the number (0, 1, or 2) of alleles with a particular index in a genotype implies the same partial ordering of genotype space as defined in Equation 1. Thus, our proposed definition of monotonicity of GP maps, and in particular the use of isotonic regression to quantify monotonicity, may be viewed as a relaxation of the linearity assumption underlying current quantitative genetics theory. In this perspective the positive correlation between monotonicity and additivity (Figure 3) is expected.

We have addressed GP maps with 2 and 3 loci as we considered an in-depth study of the properties of GP maps with higher number of loci to be outside the scope of this study. Some general observations can be made, though. Since *m* is a weighted average, the *m*_*k*_ of major loci (i.e., for which *T*_*k*_ is large relative to ∑ *T*_*k*_) will tend to dominate. For instance, in a case with a single major locus showing monotone gene action and several minor loci showing order-breaking, the GP map will overall be close to monotone (*m* close to 1). Conversely, order-preservation in a number of minor loci would have little influence on *m* if major loci have strongly non-monotone gene action. Isotonic regression gives an overall measure of monotonicity of a GP map, but provides no locus-specific measures corresponding to *m*_*k*_. Similar to the case for *m*, the gene action of major loci will have high influence on the value of *R*^2^_mono_.

The observation that monotonicity is an important property of GP maps is in principle not new. For a single locus, non-monotone gene action appears in the form of over- or under-dominance, while complete and partial dominance as well as additivity exemplify monotone gene action. Weinreich et al. (2005) distinguished between *sign epistasis* and *magnitude epistasis* and showed that sign epistasis limits the number of mutational trajectories to higher fitness. As sign epistasis reflects a non-monotone GP relationship and magnitude epistasis reflects a monotone one, this insight concords with our results. A similar distinction has been proposed (Wang et al., 2010) for statistical interactions where *removable interactions* are those that can be removed by a monotone transformation of the phenotype scale, while non-monotonicity in the GP map leads to *essential interactions*. Wu et al. (2009) developed a method to screen for and test the significance of essential interaction in genome-wide association studies. Isotonic regression has also recently been applied to link genotype and phenotype data (Beerenwinkel et al., 2011; Luss et al., 2012). Our treatment of monotonicity is more general than these earlier works in three major ways. First, we deal with monotonicity of the GP map as a whole rather than either intra-locus (dominance vs. overdominance) or inter-locus (magnitude vs. sign epistasis and removable vs. essential interactions). Second, where the earlier treatments have focused on classifying the type of gene action, we make use of quantitative measures of monotonicity. Third, our approach combining the concept of monotonicity with cGP models opens a direct link between genetics and the theory of dynamical systems in the wide sense.

Monotonicity is a property of the GP map separate from the allele frequencies, making it a physiological (Cheverud and Routman, 1995) or functional (Hansen and Wagner, 2001) descriptor rather than a statistical one. The distinction between physiological and statistical epistasis has lead to much debate (Phillips, 2008). Zeng et al. (2005) argued the distinction was unnecessary and potentially misleading. Although their arguments around orthogonality and variance components are valid, our results demonstrate very clearly that describing the properties of the GP map without reference to any particular study population is essential if we want to connect quantitative genetics with regulatory biology.

It is clear from our results that positive feedback and incoherent feedforward promote non-monotonicity. The clear-cut differences in monotonicity between different classes of regulatory networks, combined with the strong correlation between monotonicity and additivity of GP maps, appear therefore to explain the findings that regulatory systems with positive feedback give considerably more statistical epistasis than those without (Gjuvsland et al., 2007; Wang et al., 2013). Even though both incoherent feedforward and positive feedback predispose for non-monotone GP maps, the underlying mechanisms are different for the two regulatory motifs. In the case of incoherent feedforward the sum of direct and indirect effects may result in a non-monotone dose-response relationship (Kaplan et al., 2008). That positive feedback loops can give non-monotonicity is intuitively less clear, but in the Appendix we show both results analytically. Positive feedback predisposes for multiple steady states, and order-breaking might also emerge from different genotypes corresponding to different states. It should be noted, however that positive feedback is only a necessary condition for multistationarity (Plahte et al., 1995), and a positive loop in the connectivity matrix *A* of a system is not necessarily active at any point during the time course of the system.

Without any restrictions on the connectivity of a three-gene system there are 3^9^ = 19, 683 possible distinct networks. The main restriction we imposed (see Models and Methods for details) was a maximum of two regulators per gene, which allowed us to use Boolean gene regulation functions already established in the sigmoid formalism (Plahte et al., 1998). Other model formalisms allowing an arbitrary number of regulators are also available (Wagner, 1994, 1996; Siegal and Bergman, 2002) and could be extended to diploid forms and used in later studies.

Although this study has focused on gene regulatory networks, the concept of monotone gene action applies to the propagation of genetic variation across the whole physiological hierarchy. One may therefore systematically use the concepts and methods presented here to study the order-preserving and order-breaking properties of genotype-phenotype mappings that are associated with any regulatory structure amenable for mathematical modeling. Through this it will be possible to make a wide-ranging survey of which regulatory anatomies promote monotonicity and which promote non-monotonicity. We foresee that this classification may become instrumental for predicting how phenotypic effects of genetic variation propagate across generations in sexually reproducing populations.

### Conflict of interest statement

The authors declare that the research was conducted in the absence of any commercial or financial relationships that could be construed as a potential conflict of interest.

## Supplementary material

Spreadsheet S1 **Excel spreadsheet with connectivity matrices and loop products for all 1881 gene regulatory networks**.

## Appendix

In this appendix we complement the simulation studies in the main text with some analytic results for GP maps emerging from ODE models of gene regulatory networks. We study a generalization of the gene network model in Equation (3) with an arbitrary number of loci and monotone gene regulation functions, but restrict the analysis to genotype-parameter maps without pleiotropy. In particular, we show that (i) if there are no positive feedback loops and no incoherent feedforward loops in the network, the resulting GP maps are always monotone, (ii) a positive feedback loop or an incoherent feedforward loop may lead to non-monotone GP maps. The results hold for phenotypes given as the stable concentration of the product of one of the genes, and under certain restrictions also for phenotypes given as a function of one or several stable gene product concentrations that is monotonic with respect to each of its arguments.

### Gene network model

We consider a dynamic system consisting of *n* mutually interacting diploid loci *X*_*j*_, *j* ∈ *N* = {1, …, *n*}, regulating each other's expression. The time dependent output of *X*_*j*_ is denoted *z*_*j*_, and we define *z* = [*z*_1_, *z*_2_, …, *z*_*n*_]. It goes without saying that *z*_*j*_ in general depends on the genotypes of all the genes even though we will not always state this explicitly.

For a given genotype *g* = *g*_*j*_*g*^(*j*)^ = *a*_*j*_*b*_*j*_*g*^(*j*)^, where *g*_*j*_ ∈ {11, 12, 22} denotes the genotype and *a*_*j*_, *b*_*j*_ ∈ 1, 2 denote the indexes of the two alleles of locus *X*_*j*_, the equations of motion for *X*_*j*_ are

(A1) $$\begin{array}{l} {\dot{z}}_{j}^{1}=\alpha_{j}^{a_{j}}r_{j}^{a_{j}}(z)-\gamma_{j}^{a_{j}}z_{j}^{1},\\ {\dot{z}}_{j}^{2}=\alpha_{j}^{b_{j}}r_{j}^{b_{j}}(z)-\gamma_{j}^{b_{j}}z_{j}^{2},\\ z_{j}\;=z_{j}^{1}+z_{j}^{2}, \end{array}$$

where *z*^1^_*j*_ and *z*^2^_*j*_ are the time-dependent outputs of the two homologous copies of *X*_*j*_. The two allele rate functions *r*^1^_*j*_(*z*) and *r*^2^_*j*_(*z*) have range [0, 1] so that α^1^_*j*_ and α^2^_*j*_ represent the maximum production rates of the two alleles. We assume that all dose-response functions in Equation (A1) are differentiable and monotonic with respect to each of its arguments, and that for each *j, k*, the signs of ∂*r*^1^_*j*_/∂*x*_*k*_ and ∂*r*^2^_*j*_/∂*x*_*k*_ in the stable point *x* are equal. This model generalizes Eq. (3) to an arbitrary number of loci and a broader class of gene regulation functions.

In the following we are only concerned with the steady states of Equation (A1), and assume for simplicity that they have just a single stable equilibrium point. Solving the equilibrium conditions of Equation (A1) with respect to *z*^1^_*j*_ and *z*^2^_*j*_ and adding gives

(A2) $$f_{j}(x)=\mu_{j}^{a_{j}}r_{i}^{a_{j}}(x)+\mu_{j}^{b_{j}}r_{j}^{b_{j}}(x)-x_{j}=0,\;j\in N,$$

where *x* = [*x*_1_, …, *x*_*n*_] is the stable point, μ^*a*_*i*_^_*j*_ = α^*a*_*j*_^_*j*_/γ^*a*_*j*_^_*j*_ and μ^*b*_*j*_^_*j*_ = α^*b*_*j*_^_*j*_/γ^*b*_*j*_^_*j*_. Since our definition of monotonicity of GP maps does not depend on the numbering of alleles, we will without loss of generality assume μ^1^_*j*_ ≤ μ^2^_*j*_ for all *j*.

The network architecture can be read out from the structure of the system's Jacobian matrix in the stable state *x*. We define the elements of the Jacobian *J* for the set of functions *f*_*j*_ defined in Equation (A2) by

(A3) $$J_{jk}=J_{jk}(g)=\frac{\partial f_{j}(x)}{\partial x_{k}},\;j,k\in N.$$

To the Jacobian *J* it is customary to assign a signed directed graph  in which each locus *X*_*k*_ is represented by a node *X*_*k*_, and in which there is an arc from *X*_*j*_ to *X*_*k*_ if and only if *J*_*kj*_ ≠ 0, its sign given by the sign of *J*_*kj*_. A chain from *X*_*j*_ to *X*_*k*_ is a set of arcs in  leading from *X*_*j*_ to *X*_*k*_ in which all intermediate nodes are visited only once. The sign of a chain is equal to the product of the signs of the *J*_*ij*_ corresponding to the arcs in the chain. If there is a chain from *X*_*i*_ to *X*_*j*_ and also a chain from *X*_*j*_ to *X*_*i*_ through a disjoint set of nodes, the two chains constitute a proper feedback loop (FBL). To each FBL is associated a loop product *L* which is the product of the Jacobian elements corresponding to all the arcs in the loop. The sign of the loop is given by the sign of *L*. Two chains from *X*_*j*_ to *X*_*i*_, *i* ≠ *j*, with only the endpoint nodes in common, constitute a feedforward loop (FFL). If the two chains have opposite signs, the FFL is incoherent (IFFL), otherwise it is coherent (CFFL).

The system's phenotype could be any scalar quantity defined by its equilibrium value *x*. In the following we assume the genotype-phenotype map *G*(*g*) = *x*_*q*_(*g*), *q* ∈ *N*, for a given and fixed *q*, and investigate the monotonicity properties of *G*(*g*_*k*_*g*^(*k*)^) with respect to genetic variation in any locus *X*_*k*_ for different backgrounds *g*^(*k*)^. In the following sections we analyse the causes of order-breaking in *G* in the restricted case in which there is only genetic variation in μ^1^_*k*_ and μ^2^_*k*_, not in the shape of the dose-response functions *r*^1^_*k*_ and *r*^2^_*k*_, implying *r*^1^_*k*_(*x*) = *r*^2^_*k*_(*x*) = *r*_*k*_(*x*). This is what we mean by a genotype-to-parameter map without pleoitropy.

In the next sections we prove the following result:

**Proposition 1**. *Assume all rate functions in Equation (A1) are monotonic and that G is the mapping from g to x_q_ for some fixed q so that x_q_(g) is the phenotype. If there is no feedback loop (FBL) and no feedforward loop (FFL) anywhere in the network corresponding to the system Equation (A1), then necessarily m_k_ = 1 for all k. If the system contains either a single FFL or a single FBL, then G may be non-monotone for some x_k_ if the FFL is positive or the FBL is incoherent, but if the FBL is negative or the FFL is coherent, no order breaking can occur for any x_k_*.

At the end of this note we show that under some reasonable conditions this result is also valid for more general phenotypes depending on more than one *x*_*q*_.

### Networks without loops

We first consider networks containing no feedforward loop and no feedback loop. In these networks there is at most one chain from one node to another, and of course no autoregulatory loops. If there is a chain from *X*_*j*_ to *X*_*k*_, there is no chain from *X*_*k*_ to *X*_*j*_. Any node is either unregulated (constitutively expressed) or regulated by one or several other nodes.

We first prove a useful lemma.

**Lemma 1**. *If x_l_(11g^(j)^) ≤ x_l_(12g^(j)^) ≤ x_l_(22g^(j)^) for any j and l and there is an arc X_l_ → X_m_ with positive sign and no other chain from X_l_ → X_m_, then also x_m_(11g^(j)^) ≤ x_m_(12g^(j)^) ≤ x_m_(22g^(j)^). If the sign of the arc is negative, then x_m_(11g^(j)^) ≥ x_m_(12g^(j)^) ≥ x_m_(22g^(j)^).*

*Proof*. Suppressing the explicit dependence on other genes that are not affected by genetic variation in *X*_*j*_, we have

(A4) $$\begin{array}{l} x_{m}(11g^{\left(j\right)})=2\mu_{m}^{1}r_{m}(x_{l}(11g^{\left(j\right)})),\\ x_{m}(12g^{\left(j\right)})=(\mu_{m}^{1}+\mu_{m}^{2})r_{m}(x_{l}(12g^{\left(j\right)})),\\ x_{m}(22g^{\left(j\right)})=2\mu_{m}^{2}r_{m}(x_{l}(22g^{\left(j\right)})). \end{array}$$

Now, *r*_*m*_ is monotonic by assumption. If it is monotonically increasing,

(A5) $$\begin{array}{l} x_{m}(12g^{\left(j\right)})\geq (\mu_{m}^{1}+\mu_{m}^{2})r_{m}(x_{l}(11g^{\left(j\right)}))\geq x_{m}(11g^{\left(j\right)}),\\ x_{m}(22g^{\left(j\right)})\;\geq 2\mu_{m}^{2}r_{m}(x_{l}(12g^{\left(j\right)}))\geq x_{m}(12g^{\left(j\right)}), \end{array}$$

from which the assertion follows. If *r*_*m*_ is monotonically decreasing, we find the same relations with the inequality signs reversed.        □

If there is no chain from *X*_*j*_ to *X*_*q*_, genetic variation in *X*_*j*_ will not be reflected in *G*, i.e. *G*(11*g*^(*j*)^) = *G*(12*g*^(*j*)^) = *G*(22*g*^(*j*)^), and by definition does not give order-breaking. Then assume *X*_*j*_ is upstream relative to *X*_*q*_ and that the chain from *X*_*j*_ to *X*_*q*_ is positive. We first let *X*_*j*_ be an unregulated node with no predecessor. Then

(A6) $$\begin{array}{l} x_{j}(11g^{\left(j\right)})=2\mu_{j}^{1},\\ x_{j}(12g^{\left(j\right)})=\mu_{j}^{1}+\mu_{j}^{2},\\ x_{j}(22g^{\left(j\right)})=2\mu_{j}^{2}, \end{array}$$

because *r*^1^_*j*_ = *r*^2^_*j*_ = 1. From this it follows that *x*_*j*_(11*g*^(*j*)^) ≤ *x*_*j*_(12*g*^(*j*)^) ≤ *x*_*j*_(22*g*^(*j*)^).

Repeated use of Lemma 1 leads eventually to *x*_*q*_(11*g*^(*j*)^) ≤ *x*_*q*_(12*g*^(*j*)^) ≤ *x*_*q*_(22*g*^(*j*)^), irrespective of the genotypic background of *X*_*j*_. If the chain from *X*_*j*_ to *X*_*q*_ is negative, the argument goes in the same way, but then *x*_*q*_(11*g*^(*j*)^) ≥ *x*_*q*_(12*g*^(*j*)^) ≥ *x*_*q*_(22*g*^(*j*)^). The above argument can be carried out in the same way if *X*_*j*_ is not top-stream. It follows that in a network without FFBs and FFLs and where genetic variation is restricted to μ^1^_*k*_ and μ^2^_*k*_, the genotype-phenotype map *G*(*g*) = *x*_*q*_(*g*) cannot be order-breaking.

### Networks with a feedback loop

In this section we investigate the effects of feedback loops on the degree of monotonicity. Assuming monotonic dose-response functions and non-pleiotropic genetic variation, we show that a positive feedback loop may lead to order breaking, while negative feedback loops never do. We consider a network in which there is no FFL and a single FBL with *X*_*q*_ as one of its members and *X*_*k*_ is upstream of the loop.

**Lemma 2**. *Consider a network with n nodes for which all dose-response functions are monotonic and there is only genetic variation in μ^1^_k_ and μ^2^_k_. Asssume there is a chain from X_k_ to X_1_, that X_1_, but not X_k_, is member of a FBL with m nodes, and that there is no other FBL and no FFL in the system. If X_q_ is in the loop, let the loop be X_1_ → X_2_ → … → X_q_ → … → X_m_ → X_1_. If the FBL is positive, there may be order-breaking in X_q_ due to genetic variation in X_k_, but no order-breaking can occur if the loop is negative. If X_q_ is downstream of the loop, the same result applies.*

*Proof*. With a single FBL and no FFL there is at most one directed path from any node *X*_*i*_ to any other node *X*_*j*_, and if there is a path from *X*_*i*_ to *X*_*j*_, there is no return path from *X*_*j*_ to *X*_*i*_ if either *X*_*i*_ or *X*_*j*_ is not part of the FBL. We first consider the dependence of *x*_1_ on *x*_*k*_. The direct regulators of node *X*_1_ are *X*_*m*_ and *X*_*l*_, the latter being the last but one node in the chain from *X*_*k*_ to *X*_1_. In Plahte et al. (2013) we introduced *the propagation functions x*_*j*_ = *p*_*jk*_(*x*_*k*_) which express the effect on *x*_*j*_ of genetic variation in *X*_*k*_. An important property of *p*_*jk*_ is that it can be derived from all the equilibrium conditions Equation (A2) except the equation for *f*_*k*_. This implies that the effects on *X*_*j*_ of genotypic variation in *X*_*k*_ are only expressed in terms of the variations in *x*_*k*_, while the parameters expressing the genotype of *X*_*k*_ do not enter into the function *p*_*jk*_.

We then have *x*_*l*_ = *p*_*lk*_(*x*_*k*_) and *x*_*m*_ = *p*_*m*1_(*x*_1_). To make it easier to use the results in Plahte et al. (2013) we rewrite the equilibrium condition Equation (A2) as

(A7) $$R_{j}(x)-\gamma_{j}x_{j}=0,$$

where γ_*j*_ > 0. In the following, the Jacobian refers to this set of equations, which has the same root and the same functional dependencies between the variables as the original set. The signs of the partial derivatives of *R*_*j*_ are the same as for *r*^*a*_*j*_^_*j*_ and *r*^*b*_*j*_^_*j*_. The equilibrium condition for *X*_1_ is then

(A8) $$\gamma_{1}x_{1}=R_{1}(p_{lk}(x_{k}),p_{m1}(x_{1}))).$$

This equation defines *x*_1_ as a function of *x*_*k*_ in an open domain around the equilibrium point and with a derivative that can be computed by implicit differentiation, i.e.

(A9) $$\gamma_{1}\frac{\text{d}x_{1}}{\text{d}x_{k}}=\frac{\partial R_{1}}{\partial x_{l}}q_{lk}+\frac{\partial R_{1}}{\partial x_{m}}q_{m1}\frac{\text{d}x_{1}}{\text{d}x_{k}},$$

where *q*_*ij*_ = *p*′_*ij*_ is the derivative of *p*_*ij*_ for all *i, j*.

From Lemma 1 it follows that there is no order breaking in *X*_*l*_, in other words, *q*_*lk*_ has a fixed sign. Consider then *q*_*m1*_. There is just a single chain from *X*_1_ to *X*_*m*_, and Equation (13) in Plahte et al. (2013) gives

(A10) $$q_{m1}(x_{1})={\left(-1\right)}^{m\;-\;1}\frac{D_{VV}C_{U}}{D^{\left(11\right)}}.$$

Here *U* is the set of nodes in this chain, *C*_*U*_ is its chain product, i.e. the product of the Jacobian elements corresponding to the arcs in the chain, *V* = *N* \ *U, D*^(11)^ is the subdeterminant of *J* with row 1 and column 1 deleted, and *D*_*VV*_ is the subdeterminant of *J* composed of the rows and columns *V*. Because there is no feedback loop among the nodes represented in *D*^(11)^ and *D*_*VV*_, only the diagonal degradation terms contribute to these two determinants. Hence *D*^(11)^ = (−1)^*n* − 1^ ∏_*i* ≠ 1_ γ_*i*_. Similarly, *D*_*VV*_ = (−1)^*n* − *m*^ ∏_*i* ∈ *V*_ γ_*i*_, giving *q*_*ml*_ = γ_1_*C*_*U*_/Γ_*U*_, where Γ_*U*_ = ∏_*i* ∈ *U*_ γ_*i*_. Finally, we note that *P* = (∂ *R*_1_/∂ *x*_*m*_)*C*_*U*_ is the loop product of the loop.

Solving Equation (A9) with respect to d*x*_1_/d*x*_*k*_ and using all these expressions lead to

(A11) $$\gamma_{1}\frac{\text{d}x_{1}}{\text{d}x_{k}}=\frac{\Gamma_{U}}{\Gamma_{U}-P}\frac{\partial x_{1}}{\partial x_{l}}q_{1k}.$$

The sign of ∂*x*_1_/∂*x*_*l*_ is independent of the genotype of *X*_*k*_ and the sign of *q*_1*k*_ is fixed. Genotypic variation in *X*_*k*_ may change the magnitude of *P*, but its sign is fixed because all Jacobi elements have fixed sign independent of the system parameters. Thus, genotypic variation in *X*_*k*_ does not alter the sign of d*x*_1_/d*x*_*k*_ if the loop is negative (*P* < 0), while for a positive loop the sign of Γ_*U*_ − *P* may switch. In the latter case, an increase in *x*_*k*_ due to genetic variation in *X*_*k*_ may increase *x*_1_ in some cases and decrease it in others, leading to order breaking. As there is only a single chain from *X*_1_ to *X*_*q*_, no order breaking in *X*_1_ implies no order breaking in *X*_*q*_, while order breaking in *X*_1_ may propagate to *X*_*q*_. The same result follows if *X*_*q*_ is downstream a node in the loop because order breaking in this node may propagate to *X*_*q*_.    □

### Feedforward loops (FFLs)

A feedforward loop (FFL) is a motif in the network in which there are two different chains *C*_1_ and *C*_2_ from one particular node to another particular node. To each chain *C*_*i*_ is associated a chain product *P*_*i*_ defined as the product of the Jacobian elements corresponding to the arcs in *C*_*i*_. If *P*_1_ and *P*_2_ have equal signs, the FFL is coherent, otherwise it is incoherent.

In a network with a single feedforward loop and no feedback loops we now investigate the effect on *G*(*g*) = *x*_*q*_(*x*_*k*_(*g*)) of genetic variation in *X*_*k*_ for varying background *g*^(*k*)^. Our starting point is again Equation (A7). We first let *X*_*k*_ and *X*_*q*_ be the initial and terminal nodes in the FFL. The two chains *C*_1_ and *C*_2_ leading from *X*_*k*_ to *X*_*q*_ comprise ρ_1_ and ρ_2_ nodes including *X*_*k*_ and *X*_*q*_, respectively. Let the set of nodes in *C*_1_ and *C*_2_ be *X*_*U*_1__ and *X*_*U*_2__, respectively, where *U*_1_ and *U*_2_ are the corresponding subsets of *N*, and let *V*_1_ and *V*_2_ be their complements.

Roughly speaking, the derivative of the propagation function *p*_*qk*_(*x*_*k*_) can be expressed as a sum of terms, each term corresponding to one of the chains leading from *X*_*k*_ to *X*_*q*_ (Plahte et al., 2013). To the chain *C*_*i*_ is assigned the chain weight *w*_*i*_ given by

(A12) $$w_{i}={\left(-1\right)}^{\rho_{i}-1}\frac{D_{V_{i}V_{i}}}{D^{\left(kk\right)}},\;i=1,2,$$

where *D*_*V*_*i*_*V*_*i*__ is the Jacobian subdeterminant for the nodes not included in *C*_*i*_, and *D*^(*kk*)^ is the Jacobian subdeterminant for all nodes except *X*_*k*_. Because there are two chains from *X*_*k*_ to *X*_*q*_, the derivative of *p*_*qk*_ is a sum of two terms:

(A13) $$\frac{\text{d}p_{qk}}{\text{d}x_{k}}=w_{1}P_{1}+w_{2}P_{2},$$

where *P*_1_ and *P*_2_ are the two chain products, and *w*_1_ and *w*_2_ their weights (Plahte et al., 2013). When there is no feedback loop in the system, only the diagonal elements in *J* stemming from the term −γ_*i*_ *x*_*i*_ in Equation (A7) contribute to the determinants *D*_*V*_*i*_*V*_*i*__ and *D*^(*kk*)^:

(A14) $$\begin{array}{l} D_{V_{i}V_{i}}={\left(-1\right)}^{n-\rho_{i}}\prod_{j\in V_{i}}\gamma_{j},\\ D^{\left(kk\right)}={\left(-1\right)}^{n-1}\prod_{j\neq k}\gamma_{j}. \end{array}$$

Altogether this gives

(A15) $$\frac{\text{d}x_{q}}{\text{d}x_{k}}=\frac{\text{d}p_{qk}}{\text{d}x_{k}}=\frac{\gamma_{k}}{\Gamma_{1}}P_{1}+\frac{\gamma_{k}}{\Gamma_{2}}P_{2},$$

where Γ_1_ and Γ_2_ are the products of the γ_*j*_ in the two chains, respectively. The chain products *P*_1_ and *P*_2_ depend on the genotype *g*_*k*_ of *X*_*k*_ as well as on the genotypic background *g*^(*k*)^, but their signs *S*_1_ and *S*_2_ are invariant under genotypic variation. It is easy to see that a negative autoregulatory loop, which is a common feature in gene regulatory networks, would not invalidate the conclusion, but a positive autoregulatory loop might.

If the FFL is incoherent, *P*_1_ and *P*_2_ have opposite signs, implying that the sign of d*x*_*q*_/d*x*_*k*_ may vary. If the FFL is coherent, however, no order-breaking can occur.

If *X*_*k*_ is upstream relative to the initial node *X*_init_ of the FFL, it follows from the above section on networks without loops that there will be no order-breaking in *X*_init_, and the above argument is still valid.

### More general phenotypes

In real life, relevant phenotypes are not direct gene products, but rather functions of the concentrations of one or several gene products. Let the phenotype *G*(*g*) be a function of *x*_*U*_(*g*), *G* = *h*(*x*_*U*_(*g*)), where *U* is a subset of *N*, and assume that for any *u* ∈ *U*, ∂*h*/∂*x*_*u*_ has fixed sign for all genotypes. To analyse this case we extend the original system Equation (A2) to

(A16) $$\begin{array}{l} \mu_{i}^{a_{i}}r_{i}^{a_{i}}(x(g))+\mu_{i}^{b_{i}}r_{i}^{b_{i}}(x(g))-x_{i}(g)=0,\;i=1,\ldots ,n,\\ h(x_{U}(g))-x_{n\;+\;1}=0, \end{array}$$

and apply our above results to this system, in which *G*(*g*) = *x*_*n* + 1_(*g*), i.e. *q* = *n* + 1. If there are two nodes among *X*_*U*_ which have a common predecessor *X*_*k*_, then there will exist two chains from *X*_*k*_ to *X*_*n* + 1_. These two chains constitute a feedforward loop with *X*_*n* + 1_ as final node. If this FFL is incoherent, order breaking due to genetic variation in *X*_*k*_ may occur even if there is no order breaking in the original system comprising the nodes *X*_1_, …, *X*_*n*_. If the FFL is coherent, order breaking only occurs if it occurs in the original system.
